# Porcine Epidemic Diarrhea Virus Replication in Duck Intestinal Cell Line

**DOI:** 10.3201/eid2103.141658

**Published:** 2015-03

**Authors:** Mahesh Khatri

**Affiliations:** Ohio State University, Wooster, Ohio, USA

**Keywords:** PEDV, porcine epidemic diarrhea virus, intestinal epithelial cells, PEDV replication, swine, duck intestinal epithelial cell line, viruses

**To the Editor:** Porcine epidemic diarrhea virus (PEDV) was first detected in pigs in the United States in May 2013 (*1*). Since then, according to the American Association of Swine Veterinarians (https://www.aasv.org, see link to number of new cases reported), PEDV has spread to 41 states, and as of October 15, 2014, 8,622 confirmed cases of PEDV infection have been reported in swine. PEDV (family *Coronaviridae,* genus *Alphacoronavirus*) is an enveloped, positive-sense, single-stranded RNA virus (*2*). The virus replicates in epithelial cells of small and large intestines and causes highly contagious infection in pigs. The disease is characterized by watery diarrhea, vomiting (leading to subsequent dehydration), and high rates of death, especially in young piglets; thus, outbreaks cause substantial economic losses to the swine industry (*1*). Variants of the original virulent PEDV have recently been isolated in the United States, making development of a vaccine to protect against this devastating disease even more challenging (*3*). Vero cells are used for the isolation of virus from clinical samples and for virus propagation and titration and virus neutralization studies. The addition of exogenous trypsin in culture medium is a prerequisite for efficient replication of PEDV in Vero cells (*4*): trypsin cleaves the spike protein of PEDV into 2 subunits that mediate cell-to-cell fusion and virus entry into the cells (*5*).

We examined PEDV replication in a newly established immortalized duck intestinal epithelial cell (MK-DIEC) line, which was generated from the intestinal tissues of a 19- day-old white Pekin duck embryo. MK-DIECs are cuboidal (characteristic of epithelial cells), express epithelial marker (pan-cytokeratin), and show extensive proliferation in culture. Several coronaviruses, including PEDV, use aminopeptidase N (APN) as the cellular receptor for attachment to cells (*6*). As a first step, we used a rabbit polyclonal anti-human APN antibody (Abcam, Cambridge, MA, USA) in an indirect immunofluorescence assay (IFA) to examine whether MK-DIECs express APN. We found that nearly 100% of the cells expressed APN on their surface (Technical Appendix Figure 1).

Next, we examined PEDV replication in MK-DIECs. The cells were cultured in medium containing equal amounts of Dulbecco modified Eagle medium; Mammary Epithelial Growth Medium (Lonza, Walkersville, MD, USA) supplemented with bovine pituitary extract (70 μg/mL), human epidermal growth factor (5 ng/mL), insulin (5 μg/mL), and hydrocortisone (0.5 μg/mL); and 2% fetal bovine serum. Near confluent cells were infected with PEDV at a multiplicity of infection of 0.1. The Colorado strain of PEDV (obtained from the National Veterinary Services Laboratories, Ames, IA, USA), which was initially passaged 5 times in Vero cells, was used to infect the MK-DIECs. After adsorption for 1 h, the cells were cultured in serum-free Dulbecco modified Eagle medium supplemented with 0.02% yeast extract, 0.3% tryptose phosphate broth, and 1% penicillin/streptomycin (infection medium). To examine the requirement of trypsin (Sigma, St. Louis, MO, USA) for PEDV replication in MK-DIECs, we added trypsin (0, 2.5, 5, or 10 μg/mL) to the infection medium.

We also cultured MK-DIECs in 96-well plates and similarly infected them with PEDV for the detection of PEDV nucleoprotein (NP) by IFA using fluorescein isothiocyanate–labeled mouse PEDV NP monoclonal antibody (SD-1F; Medgene Labs, Brookings, SD, USA). At 12, 24, and 36 h after infection, released virus in infected cells was quantified by virus titration in Vero cells by inoculating 10-fold serial dilutions. After 24 h, viral NP was detected by IFA staining. The virus titer was calculated according to the Reed–Muench method and expressed as the 50% tissue culture infectious dose/mL.

We detected PEDV NP in MK-DIECs 24 hours after infection in medium with and without trypsin (data not shown). However, the numbers of cells positive for PEDV NP was larger in cells cultured with trypsin (2.5 μg/mL and 5 μg/mL) than without trypsin (Figure, panel A). PEDV also induced cytopathic effects (CPEs) in these cells, which were characterized by rounding of cells, syncytium formation, and cell detachment. The CPEs were more pronounced in cells infected with added trypsin; as little as 2.5 μg/mL of trypsin in infection medium was sufficient to induce substantial CPEs in infected cells (Figure, panel B). No signs of CPEs were observed in uninfected control cells, and cells did not display any trypsin-mediated toxicity. The virus titers were detectable in PEDV-infected cells 12 h after infection. The titers further increased at 24 h, reaching a peak at 36 h after infection. Infected cells in infection medium with 10 μg /mL added trypsin had the highest titers (Technical Appendix Figure 2).

**Figure:**
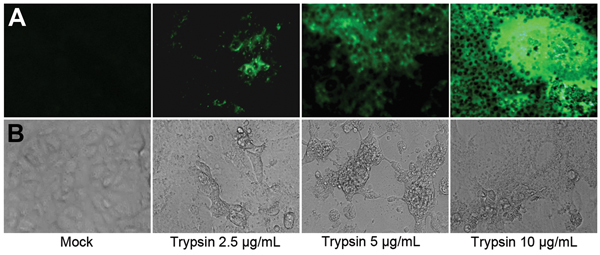
Replication of porcine epidemic diarrhea virus (PEDV) in a newly established immortalized duck intestinal epithelial cell line (MK-DIEC) infected with PEDV at a multiplicity of infection of 0.1 in the presence of different concentrations of trypsin. A) Twenty-four hours after infection, PEDV nucleoprotein in infected cells was detected by immunofluorescence assay using fluorescein isothiocyanate–labeled nucleoprotein-specific monoclonal antibody. B) PEDV-induced cytopathic effect in MK-DIEC cells 36 h after infection.

Coronaviruses are RNA viruses that are prone to high levels of mutation resulting in novel reassortants. Birds and bats are considered reservoirs of coronaviruses. However, reserviors of PEDV are not yet known. In conclusion, we have demonstrated that PEDV replicates in MK-DIECs. Availability of a cell line of intestinal origin that supports PEDV replication may be of value for studying mechanisms of virus–cell interactions and for developing live attenuated and killed vaccines.

Technical Appendix Figures showing expression of aminopeptidase N on immortalized duck intestinal epithelial cell (MK-DIEC) line and quantification of released progeny virus in supernatant of porcine epidemic diarrhea virus–infected MK-DIECs.
