# Supplementary material for: Porcine Epidemic Diarrhea Virus Replication in Duck Intestinal Cell Line
Source: Emerg Infect Dis. 2015 Mar;21(3):549–50. doi: 10.3201/eid2103.141658 (PMC4344288; doi:10.3201/eid2103.141658)
Supplement: Technical Appendix — Figures showing expression of aminopeptidase N on immortalized duck intestinal epithelial cell (MK-DIEC) line and quantification of released progeny virus in supernatant of porcine epidemic diarrhea virus–infected MK-DIECs. [file 14-1658-Techapp-s1.pdf]

DOI: http://dx.doi.org/10.3201/eid2103.141658

Porcine Epidemic Diarrhea Virus Replication in Duck Intestinal Cell Line

Technical Appendix

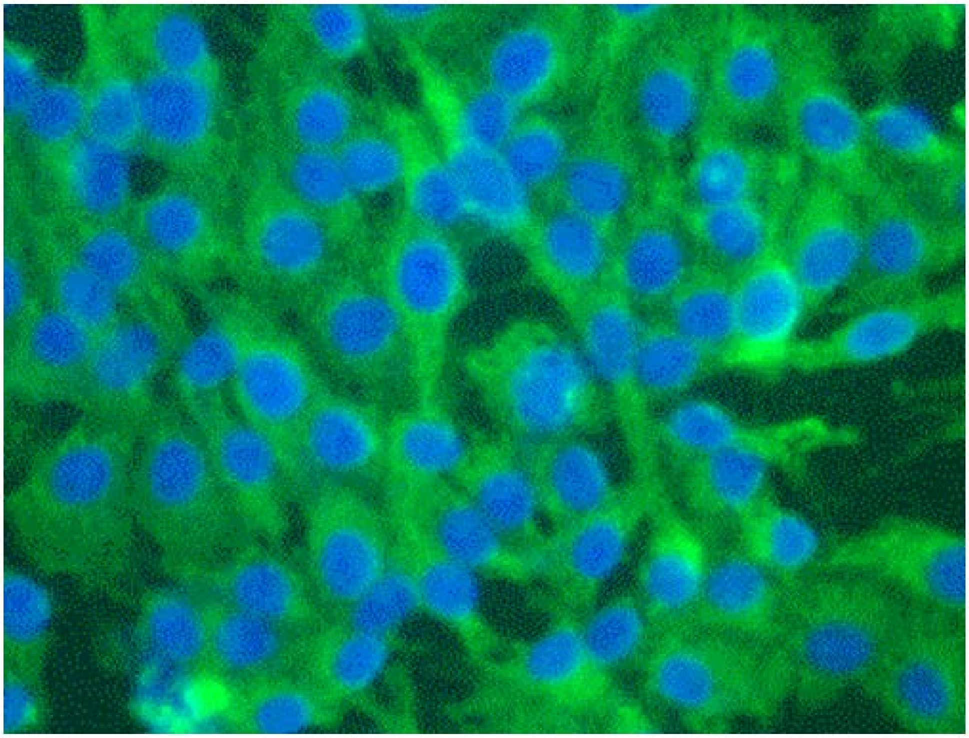

Technical Appendix Figure 1. Expression of aminopeptidase N (APN) on immortalized duck intestinal epithelial cell line (MK-DIEC). APN expression was detected on MK-DIEC cell surfaces by immunofluorescence assay using rabbit polyclonal anti-human APN as primary antibody and fluorescein isothiocyanate–labeled goat anti-rabbit as secondary antibody.

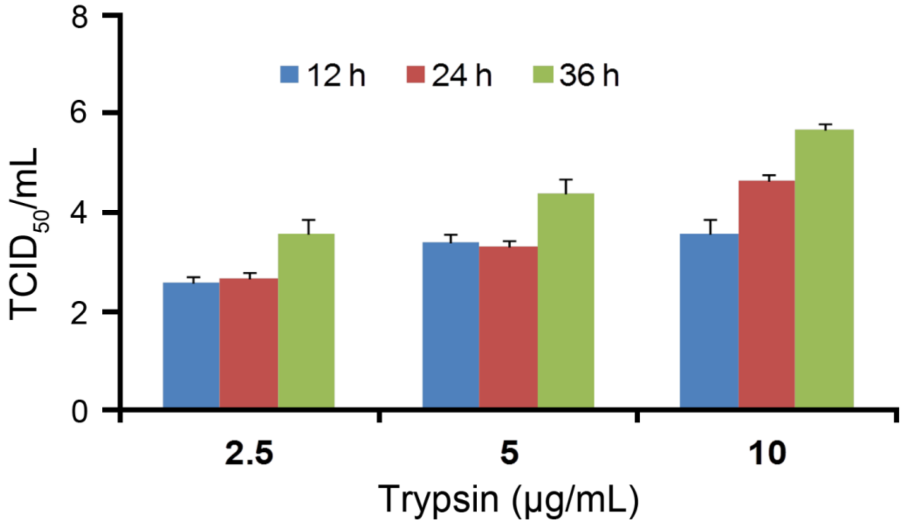

Technical Appendix Figure 2. Quantification of released progeny virus in porcine epidemic diarrhea virus–infected supernatant of immortalized duck intestinal epithelial cell line MK-DIEC cells, as measured by titration in Vero cells. Each bar represents mean (±SD) virus titer at each time point from 3 independent experiments. $TCID_{50}$, 50% tissue culture infectious dose.
